# Supplementary material for: Comorbid mental disorders and quality of life of people with epilepsy attending primary health care clinics in rural Ethiopia
Source: PLoS One. 2021 Jan 20;16(1):e0238137. doi: 10.1371/journal.pone.0238137 (PMC7817054; doi:10.1371/journal.pone.0238137)
Supplement: Attachment — Submitted filename: Response to reviewers.docx [file pone.0238137.s002.docx]

**Response to reviewers**

Thank you for the constructive comments and here is the point by point response to the editorial and reviewer comments.

Editor comments

1. Please ensure that your manuscript meets PLOS ONE's style requirements, including those for file naming.

Response 1

This has been now corrected.

2. Please include additional information regarding the survey or questionnaire used in the study and ensure that you have provided sufficient details that others could replicate the analyses.

Response 2

Additional information regarding the questionnaires used has been included and attached as supporting information (please see manuscript pages 8 and 9).

**Reviewer 1**

3. Very interesting and well conducted study about mental health issues among patients with epilepsy. However, a few minor correction will improve the manuscript and make and even better read.

Response 3: Thank you for the encouraging feedback. We hope we have addressed your comments adequately below.

4. The manuscript would clearly improve by undergoing anti-verbiage drive. It is full of unnecessary words and repetitions which could be removed without compromising the clarity of the report.

Response 4: We have edited the manuscript and attempted to remove unnecessary words and repetitions.

5. Could the authors please avoids the use of PWE as this is a matter of irritation to many patients with epilepsy, in my experience. Just spell it out in full. Also, the authors should remember that this is report on epilepsy so there is no need to repeat this at each paragraph.

Response 5: We have now corrected this throughout the manuscript.

6. The author don't seem to be cognizant with the concept of keywords. Indeed, any term or word which is already in the title should not be in the list of keywords. Could this be corrected?

Response 6: We have now corrected the keywords.

**Reviewer 2**

7. The authors present a cross-sectional study on the prevalence of mental comorbidities in persons with epilepsy from a region in Ethiopia. The study is of interest for clinicians and researchers especially as it is from an area from which only limited data is available. The two-staged approach of identifying study participants and the methods of diagnostic assessment and data collection are as well of interest. I have only a few comments:

Response 7: Thank you for your helpful comments.

8. Epilepsy duration should be taken into account.

Response 8

We have rerun the analysis including duration of epilepsy and have included the revised findings in the manuscript. This has not affected the main findings or interpretation.

9. I understand about half of the population are Muslims but the vast majority of study participants is of orthodox religion. Could the authors explain? At least they assessed for religion. Do they see any association with the study results?

Response 9

In the Gurage zone as a whole, the majority of the population are Muslims, but in the district of Sodo and the data collection sites for this study the majority of the participants are Orthodox Christians. Therefore, our study sample reflected the population from which they were drawn.

We did not include religion in the analysis. Religion is a sensitive topic in this community. Any observed association between religious affiliation and mental ill-health (which is stigmatised) could be misunderstood as being causal, even though such an association could be confounded by other variables (such as socio-economic status). As we had no a priori justification to consider religion as an important explanatory variable for this research question we therefore omitted it from the analysis.

10. Table 2 does not show any data on anxiety disorder: Could the authors explain?

Response 10

That is correct, there were no participants with anxiety disorders. This was one of the surprising findings of this study. We noted in the discussion that this may reflect the presentation of depression in this setting, which tends to also have anxiety symptoms. The psychiatric nurses may have prioritised the diagnosis of depression over that of anxiety. We have added the following to the discussion:

*“The way in which depression and anxiety manifest and the non-biomedical causal attributions of depressive/anxiety symptoms in this socio-cultural context [**43**] could have contributed to the low detection by mental health professionals applying Western diagnostic criteria [**42**]. The diagnosis and presentation of depression in Ethiopian culture appears to include a combination of anxiety, somatic and depressive symptoms rather than typical DSM criteria of depression [**44**]. This might have masked and contributed to the absence of diagnosis of anxiety disorders in this study. Psychiatry nurses could also be biased towards diagnosing more severe conditions like depression than anxiety disorders in their clinical practice.*

We have now added this as a potential limitation to the study.

*‘It is also possible that co-morbid anxiety disorders were under-diagnosed and/or categorised as depressive disorders by the clinician interviewers.’*

11. Discussion is a bit lengthy and extends to statements which are certainly common sense (poverty reduction and improved education are necessary) but their association to the study results is rather loose.
    The main surprising feature is the smaller proportion of persons with mental comorbidity in comparison to other studies. This might be discussed further. Are there any other factors rather than those discussed by the authors? Do differences in presentation of depression have any consequences for further research?

Response 11

We have now elaborated on the relatively low levels of co-morbid mental illness and noted the relevance for future research.

We have cut some of the sentences which made recommendations that went beyond our study findings.
